# High Reproductive Success Despite Queuing – Socio-Sexual Development of Males in a Complex Social Environment

**DOI:** 10.3389/fpsyg.2019.02810

**Published:** 2019-12-17

**Authors:** Alexandra M. Mutwill, Tobias D. Zimmermann, Charel Reuland, Sebastian Fuchs, Joachim Kunert, S. Helene Richter, Sylvia Kaiser, Norbert Sachser

**Affiliations:** ^1^Department of Behavioural Biology, University of Münster, Münster, Germany; ^2^Münster Graduate School of Evolution, University of Münster, Münster, Germany; ^3^Faculty of Statistics, Technical University of Dortmund, Dortmund, Germany

**Keywords:** behavioral development, reproductive success, dominance, reproductive tactic, paternity, behavioral plasticity, guinea pigs

## Abstract

The start of actual breeding in male social mammals can occur long after individuals attain sexual maturity. Mainly prevented from reproduction by older and dominant males, young males often queue until strong enough to compete for favorable social positions and, in this way, to obtain access to females. However, to what extent maturing males also apply tactics to reproduce before this time is largely unknown. Therefore, the aim of the present study was to elucidate male socio-sexual development from onset of sexual maturity through first mating success until the achievement of a stable social position in a complex social environment. For this purpose, guinea pigs were used as a model system and reproductive success of males living in large mixed-sex colonies was assessed during their first year of life. As a reference, males in a mixed-sex pair situation were examined. Pair-housed males reproduced for the first time around the onset of sexual maturity whereas colony-housed males did so much later in life and with a considerably higher variance. In colonies, reproductive success was significantly affected by dominance status. Dominance itself was age-dependent, with older males having significantly higher dominance ranks than younger males. Surprisingly, both younger and older colony-housed males attained substantial reproductive success of comparable amounts. Thus, younger males reproduced irrespective of queuing and already before reaching a high social status. This mating success of maturing males was most likely achieved via several reproductive tactics which were flexibly applied with the onset of sexual maturity. The period of socio-sexual development before a stable social position is established may, therefore, be a time during which male mammals use flexible behavioral tactics to achieve reproductive success more frequently than commonly is presumed. In addition, the findings strongly indicate that high behavioral plasticity exists well beyond sexual maturity.

## Introduction

In many social mammals, the start of effective breeding in males can occur months or even years after individuals reach sexual maturity. This includes a wide range of carnivores (e.g., lions, *Panthera leo*: Packer et al., 1991; spotted hyenas, *Crocuta crocuta*: East and Hofer, 2002; meerkats, *Suricata suricatta*: Spong et al., 2008), ungulates (e.g., red deer, *Cervus elaphus*: Clutton-Brock and Albon, 1979), rodents (e.g., wild cavies, *Cavia aperea*: Asher et al., 2008; guinea pigs, *Cavia aperea* f. *porcellus*: Sachser, 1986; striped mice, *Rhabdomys pumilio*: Schradin et al., 2009) as well as different primate species (e.g., mandrills, *Mandrillus sphinx*: Charpentier et al., 2005; mountain gorillas, *Gorilla beringei beringei*: Bradley et al., 2005). The exclusion of younger males from reproduction within a social group is mainly caused by intrasexual selection, in particular male-male competition. Especially in situations where reproductive skew is high and breeding is mostly monopolized by only one or few alpha males, younger males are prevented from mating as they are unable to compete with the stronger dominant males (Bradley et al., 2005). In general, a high social status facilitates the access to resources including mating partners (Ellis, 1995; Spong et al., 2008; Alberts, 2012). However, such alpha positions are often constrained by a certain age, size, and weight (Haley et al., 1994; Schuett, 1997; Asher et al., 2008) or specific weaponry like horns and antlers (Lincoln, 1994; Kruuk et al., 2002) that bring about the necessary fighting abilities. As a consequence, maturing males often queue until strong enough to compete successfully and thereby obtain access to mating partners (East and Hofer, 2002; Alberts et al., 2003; Sachser et al., 2011, 2013).

The duration of breeding lifespan or tenure (i.e., period between the age when a male starts to breed effectively and the age when it stops) has a large influence on lifetime reproductive success and thus fitness. Therefore, queues of short duration in males might still be paid off by later benefits of reproduction, while long-term queues might already disadvantage the young males and promote “queue-jumping” (Wiley and Rabenold, 1984; Alberts et al., 2003). Because the time until favorable social positions are reached can be rather long in many species (East and Hofer, 2002; Alberts et al., 2003), counter tactics by maturing males against reproductive suppression might be expected. The period of socio-sexual development in male social mammals, beginning from onset of sexual maturity until the establishment of a stable social position, may therefore offer more opportunities in terms of reproduction than just queuing.

The domestic guinea pig (*Cavia aperea* f. *porcellus*) is a group-living rodent with a complex social bonding and dominance system (Sachser, 1986). In the natural habitat, its ancestor the wild cavy (*Cavia aperea*) is characterized by different forms of social organization (Asher et al., 2004, 2008). At low population densities the animals live in small groups, consisting of either mixed-sex pairs or small harems. At high population densities, wild cavies form larger harems which can be associated by male satellites (Asher et al., 2008). In addition, roaming males range over the whole area without stable spatial or social associations (Asher et al., 2008). These density-dependent social organizations can also be found in a comparable way in the domestic form (Sachser, 1986). Furthermore, male guinea pigs show high developmental plasticity during socio-sexual development. In particular, based on the social environment encountered during adolescence, they form completely different adaptive reproductive tactics (Sachser et al., 2011, 2013). More specifically, males raised in mixed-sex pairs develop a high-aggressive tactic of mate defense. In contrast, males growing up in large mixed-sex colonies establish a low-aggressive adolescent phenotype that precludes costly agonistic encounters with older and dominant males (Lürzel et al., 2010, 2011a,2011b; Zimmermann et al., 2017a). This queuing of maturing males seems to be adaptive in this complex social situation (Zimmermann et al., 2017b) as colony-housed males appear unable to effectively compete for high ranking positions with other males until an age of about 7 months, when they are fully adult (Sachser, 1986). However, paternity data are required to unequivocally clarify whether colony-housed males fail to reproduce before this time or whether they apply other reproductive tactics while queuing to reach an alpha position in adulthood.

The aim of the present study was therefore to elucidate the socio-sexual development of male guinea pigs from onset of sexual maturity through first mating success until the attainment of a stable social position in full adulthood in large mixed-sex colonies. For this purpose, reproductive success of colony-housed males was assessed over this phase of life and as a reference a mixed-sex pair situation was examined. It was hypothesized that owing to the social situation, colony-housed males would reproduce for the first time later in life (hypothesis 1, H1) and show a higher variance in time of first reproduction (H2) than pair-housed males. It was further assumed that reproductive success in colonies would be affected by dominance status (H3) and that according to previous work (Sachser, 1986), dominance itself would be age-dependent. Explicitly, we expected higher dominance status (H4) as well as a higher variance of statuses (H5) in older than in younger males. Accordingly, we predicted that reproductive success would be higher in older than in younger males (H6). Proportions of multiple paternities (= litters fathered by more than one male) were expected to be higher in younger than in older males as indication for potential sneaking tactics (H7). For the same reasons it was further assumed that proportions of multiple paternities would be affected by dominance status (H8).

## Materials and Methods

### Animals and Housing Conditions

The guinea pigs used for this study were descendants of a heterogeneous shorthaired and multicolored breeding stock of 40 founder animals obtained from a professional breeder in 1975. To counteract inbreeding, individuals from other breeders were regularly crossbred into the stock. All animals were born and reared in a total of four mixed-sex colonies, each consisting of 7–12 males, 11–16 females and their pre-weaned offspring. Each colony was kept in a wooden enclosure of approximately 6 m^2^ with wood shavings on the floor and three shelters. In each group, a graduated age structure was maintained by introducing young females (21 ± 1 days of age) every 4–6 weeks and young males (29 ± 2 days of age) every 6–8 weeks. Offspring routinely were taken out of the groups at 21 (±1) days of age, and adult guinea pigs removed at an age of about 20 months. For this study, 29-(±2)-day-old males from different litters were either moved from the natal colony to one of the other colonies (colony housing) or individually placed together with a 20-to-30-day-old unfamiliar female (pair housing). Pairs were kept in wooden enclosures of 0.5 m^2^ with wood shavings on the floor and one shelter. All animals were housed under controlled conditions with 12 h:12 h light/dark cycle (lights on at 7 am) at a temperature of about 22°C and a relative humidity of about 50%. Commercial guinea pig diet (Höveler Meerschweinchenfutter 10700, Höveler Spezialfutterwerke GmbH & Co. KG, Langenfeld, Germany) and water were available *ad libitum*. Vitamin C was provided in the water twice a week. This diet was daily supplemented with hay. Date of birth was known for all animals and natural markings in fur color patterns allowed distinctive identification of each individual.

### Experimental Approach

**FIGURE 1:**
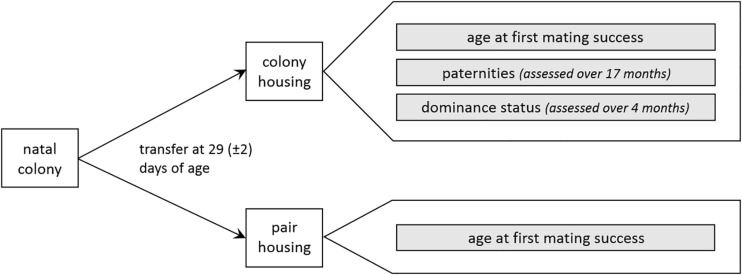
Experimental approach (for details see text).

In colony and pair housing conditions, age at first mating success was determined for all males. In colonies, paternities and dominance status were additionally assessed. An overview of the procedure is depicted in Figure 1.

#### Assessment of Mating Success

In the four colonies, paternities of the offspring were determined over a period of about 17 months. For each male the dates of mating success (i.e., when copulation resulted in fertilization) were estimated by subtracting the median gestation period of 69 days from the date of birth of respective offspring. By this means, time of first mating success could be assessed for 27 colony-housed males. In 15 pair-housed males, time of first mating success was calculated on the basis of first offspring of the partner female. For litters in colonies of which parentage could be determined for all pups, it was examined whether offspring were sired by a single male or by multiple males (= multiple paternity litters).

#### Assessment of Dominance Status

In colonies, dominance status was determined for all males of an age between 60 and 359 days over a period of about 4 months. Every focal animal was monitored live in its respective home colony for 3 × 10 min per week by use of the software The Observer XT (Observer XT 7.0, Noldus Information Technology BV, Wageningen, Netherlands). Observations were carried out in the morning and in the afternoon by a trained observer (CR) applying focal animal sampling and continuous recording (Martin and Bateson, 2007). The order of colonies and focal animals for the observations was randomized. To assess dominance status, the outcome of agonistic encounters was scored by means of retreats (= subject moves to at least one body length from opponent within 3 s following agonistic interaction or approach by an opponent). The animal provoking a retreat was regarded as the winner. On this basis, dominance index was calculated for each male by the ratio of wins to the total number of scored agonistic interactions (minimum = 5 interactions). The index ranges from 0 to 1, with high values denoting dominant and low values denoting subordinate individuals (Sachser, 1986). Reproductive success was analyzed for all males that resided in the colonies over this period of 4 months (*n* = 11) to assess whether it was affected by dominance status.

#### Assessment of Age Effects

When testing for effects of age, data of males were divided into “younger males” (60–209 days of age) and “older males” (210–359 days of age). The cut-off at 210 days was chosen as colony-housed males begin to obtain high ranking (= alpha) positions around this age (Sachser, 1986). In a first step, we analyzed whether younger (*n* = 11) and older (*n* = 12) males differed in their dominance status. For this, those males of which dominance indices were determined over at least 5 weeks were taken into account. In a second step, we examined those males observed for at least 90 days in each age group (*n* = 19) to determine age differences in reproductive success and the proportion of multiple paternities. To account for different lengths of observation periods, relative measures of reproductive success (proportion of sired to possible offspring or litters) were used for this analysis.

#### Paternity Analysis

Ear tissue samples were collected from pups as well as all potential parents and stored in 70% ethanol until analyzed. Genomic DNA was purified by first digesting the tissue samples using Proteinase K, followed by phenol/chloroform extraction and DNA precipitation with ethanol. DNA pellets were washed with 70% ethanol several times and re-suspended in TE-buffer. Fourteen microsatellites were amplified by PCR and sequenced. See Asher et al. (2008) and Kanitz et al. (2009) for further details on microsatellite loci and amplification procedures. Alleles were analyzed using GeneMarker (version 2.6.2, SoftGenetics LLC, State College, PA, United States) and all potential parents as well as all pups which were included in subsequent analyses were genotyped at a minimum of 6 loci.

Parentages were assigned at a 95% confidence level based on simulations of 100,000 cycles using the likelihood-based approach implemented in Cervus (version 3.0.7, Kalinowski et al., 2007). Allele frequency analysis of all potential parents (*n* = 212) revealed a significant deviation from Hardy–Weinberg equilibrium and an increased number of null alleles at two loci (ap13, ap16) that were hence discarded. As pups may suckle from lactating females other than their own mother under colony housing conditions, maternities could not be clearly determined by observation in some cases. Accordingly, offspring were assigned to potential parents consisting of all sexually mature males (at least 60 days of age) that resided in the respective colony at the estimated date of fertilization (±3 days) and either the known mother or up to three candidate mothers. The proportion of genotyping errors was estimated in two ways (see Hoffman and Amos, 2005): First, mismatches in repeatedly genotyped samples (*n* = 35) were counted, resulting in 18 allele mismatches in 378 loci and an error rate of 0.048 per locus. Second, known mother-offspring pairs with a single pup randomly chosen from each mother with multiple offspring (*n* = 94) were checked for allele mismatches, yielding a mean error rate across loci of 0.0078. The assigned paternities based on the more conservative error rate estimate of 0.048 per locus were chosen for all subsequent analyses.

### Statistics

From a statistical viewpoint, data were analyzed as a series of ordered hypotheses (H1–H8, see section “Introduction”). To control the familywise error-rate, null hypotheses were tested in this pre-specified order, and the (*i* + 1)-th null hypothesis was tested only if the *i*-th null hypothesis had been rejected. This so-called “gate-keeping procedure” allows testing of each of the hypotheses with the help of a level-α-test, without level adjustment, see e.g., Dimitrienko et al. (2010). Since H5 was the first hypothesis where the null hypothesis could not be rejected, we had to stop the gate-keeping procedure at this point. Hence, the gate-keeping procedure came to the conclusion that null H1, H2, H3, and H4 can be rejected, while null H5, H6, H7, and H8 have to be accepted. However, from a descriptive viewpoint, formal tests were calculated for H6–H8, to see whether there were hints of possible effects.

For analysis of first mating success (H1 and H2) a model was developed (see Supplementary Material for details) and as test statistics the Wald test (mean) and *F*-test (variance) were applied, respectively. As proposed by a referee, an additional analysis of the coefficient of variation was performed to correct for the fact that variance increases with the mean. To test the effect of dominance index on reproductive success (H3) a generalized linear model (GLM) was used on basis of a Poisson distribution. Analysis of whether younger and older individuals differed in dominance indices (H4 and H5) was performed with a Wilcoxon–Mann–Whitney test together with a Permutation test. For testing whether younger and older individuals differed in reproductive success or proportion of multiple paternities (H6 and H7) a Wilcoxon signed-rank test was used. To analyze the effect of dominance index on the proportion of multiple paternities (H8) a general linear model (LM) was applied. Our statistical analyses took account of the fact that colony-housed males lived in four different colonies.

One of the referees had reservations against the gate-keeping procedure. As an alternative, it was proposed to use the Benjamini–Hochberg procedure (Benjamini and Hochberg, 1995) to control the false discovery rate (FDR). This procedure requires that we have only one *p*-value for each hypothesis. For H3, however, we have used two possible responses. If we use the number of sired offspring, the Benjamini–Hochberg procedure (controlling the FDR at *q* = 0.05) leads to exactly the same result as the gate-keeping procedure. If we use the number of sired litters, the Benjamini-Hochberg procedure still provides a significant result for H1 and H2, while no significance can be achieved for the other hypotheses. We indicate statistical significances based on the consistent result of the gate-keeping procedure and the Benjamini–Hochberg procedure when using the number of sired offspring as a parameter for reproductive success. For a description of the detailed methods used to test the single hypotheses, see Supplementary Material.

## Results

### First Mating Success

**FIGURE 2:**
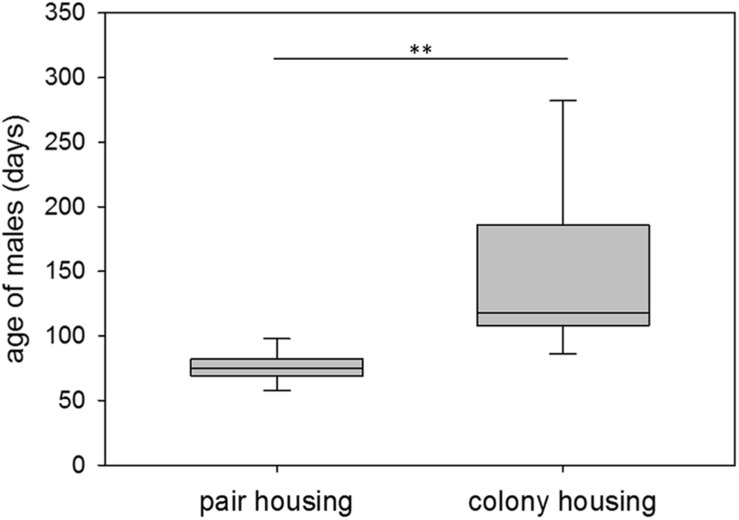
First mating success. Age of male guinea pigs (days) at first successful mating while living in pair or colony housing. Box plots represent the 25–75^th^ percentile with medians (line in box). Whisker lines represent the 10^th^ and 90^th^ percentile. Statistics: *n*_pair_ = 15, *n*_colony_ = 27; Wald test (mean): *t* = –2.90, *p* = 0.0038 (^∗∗^); *F*-test (variance): *F* = 42.23, *p* ≤ 0.0001.

At first successful mating, pair-housed males were on average 76 days old, ranging from 57 to 102 days of age. Colony-housed males sired their first offspring at 151 days of age on average with a range of 56 to 400 days of age. Hence, colony-housed males reproduced significantly later in life (*n*_pair_ = 15, *n*_colony_ = 27; Wald test: *t* = −2.90, *p* = 0.0038) and showed a significantly higher variance in the time to their first mating success than pair-housed males (*n*_pair_ = 15, *n*_colony_ = 27; *F*-test: *F* = 42.23, *p* ≤ 0.0001) (Figure 2). Subsequent testing revealed a higher coefficient of variation for colony-housed males (*n*_pair_ = 15, *n*_colony_ = 27; *F*-test: *F* = 7.98, *p* ≤ 0.0001). The larger variance in colony-housed males was therefore not explained by the larger mean alone.

### Dominance Status and Reproductive Success

**FIGURE 3:**
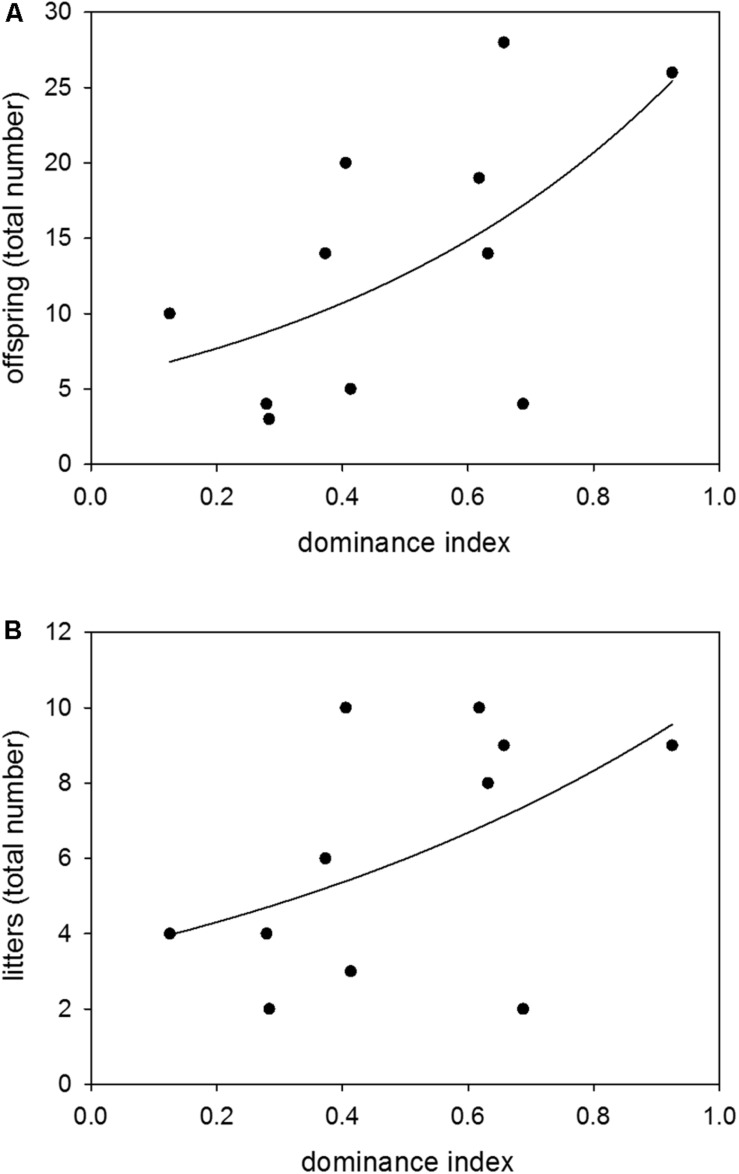
Dominance and reproduction. Effects of dominance index on **(A)** the total number of sired offspring and **(B)** the total number of sired litters. Data were based on observations of colony-housed males (60–359 days of age) over about 4 months. Statistics: *n* = 11; GLM (offspring): *z* = 4.52, *p* ≤ 0.0001; GLM (litters): *z* = 2.02, *p* = 0.0432.

Colony-housed males between 60 and 359 days of age showed a broad range of dominance indices from 0.13 to 0.93. There was a significant effect of dominance on reproductive success, with a higher dominance status leading to more sired offspring and litters (*n* = 11; GLM (offspring): *z* = 4.52, *p* ≤ 0.0001; GLM (litters): *z* = 2.02, *p* = 0.0432) (Figures 3A,B).

**FIGURE 4:**
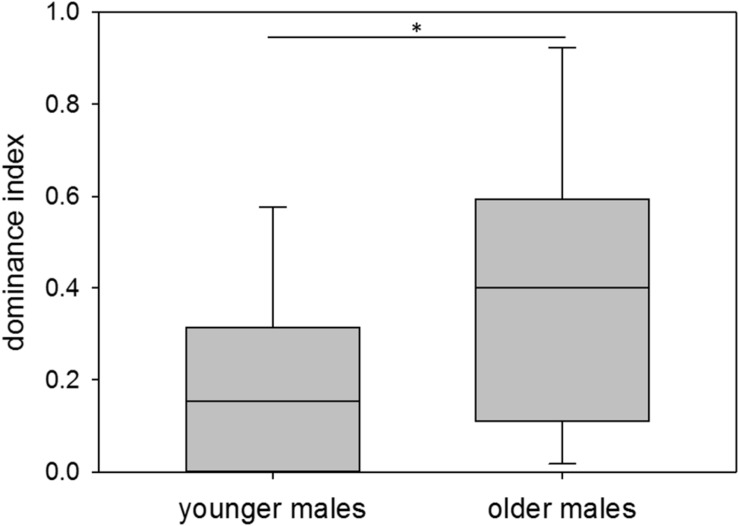
Age and dominance. Dominance indices of younger (60–209 days of age) and older (210–359 days of age) colony-housed males. Box plots represent the 25–75^th^ percentile with medians (line in box). Whisker lines represent the 10^th^ and 90^th^ percentile. Statistics: *n*_younger_ = 12, *n*_older_ = 11; Wilcoxon–Mann–Whitney test (mean): *W* = 165, *p* = 0.025 (^∗^), Permutation test: *p* = 0.0228; Permutation test (variance): *p* = 0.1605.

With respect to age, younger males showed a median dominance index of 0.15, older males a median dominance index of 0.40. Thus, younger males overall had a significantly lower dominance status than older males (*n*_younger_ = 12, *n*_older_ = 11; Wilcoxon–Mann–Whitney test: *W* = 165, *p* = 0.025; Permutation test: *p* = 0.0228) (Figure 4). Variance in dominance indices was high in both age groups, but did not differ between them (*n*_younger_ = 12, *n*_older_ = 11; Permutation test: *p* = 0.1605).

### Age and Reproductive Success

**FIGURE 5:**
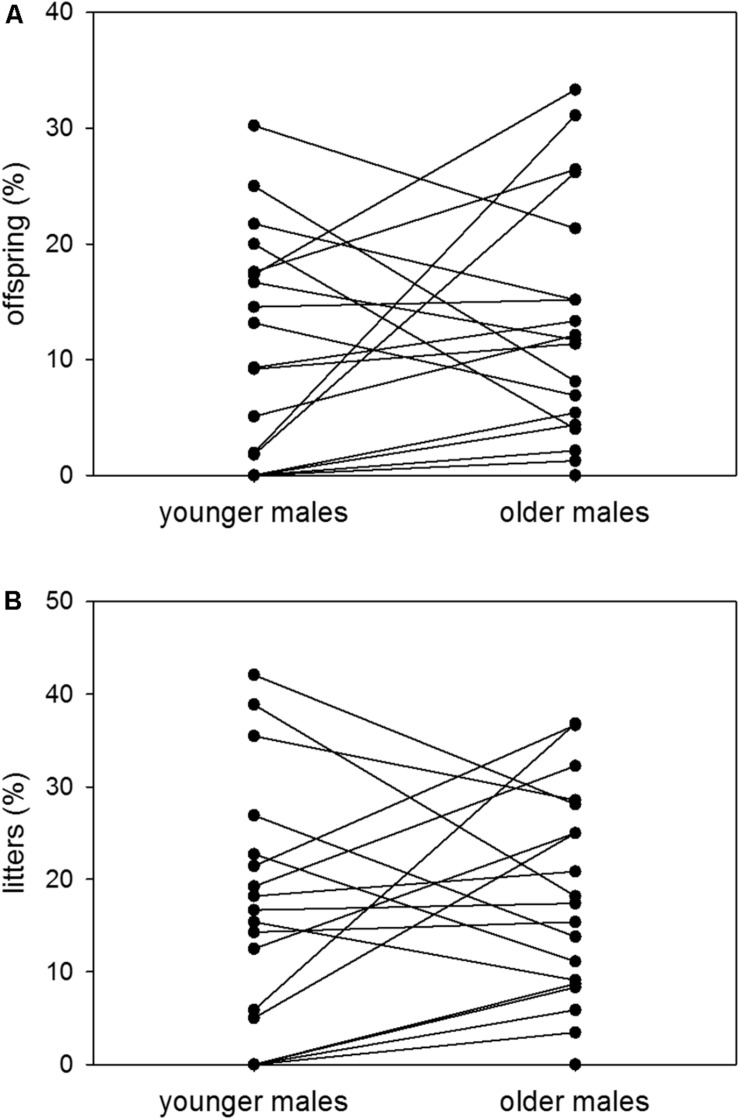
Age and reproductive success. Proportions (%) of **(A)** sired offspring to possible offspring and **(B)** sired litters to possible litters of colony-housed males in the younger (60–209 days of age) and the older (210–359 days of age) age group. Each pair of dots with connecting line represents one individual. Statistics: *n* = 19; Wilcoxon signed-rank test (offspring): *V* = 70, *p* = 0.2568; Wilcoxon signed-rank test (litters): *V* = 67, *p* = 0.2165.

At younger ages, males sired 11% of possible offspring and contributed to 16% of possible litters on average. At older ages, the same males sired 13% of possible offspring and contributed to 18% of possible litters on average. Thus, there was no difference in male reproductive success between the younger and the older age group [*n* = 19; Wilcoxon signed-rank test (offspring): *V* = 70, *p* = 0.2568; Wilcoxon signed-rank test (litters): *V* = 67, *p* = 0.2165] (Figures 5A,B).

### Multiple Paternities

**FIGURE 6:**
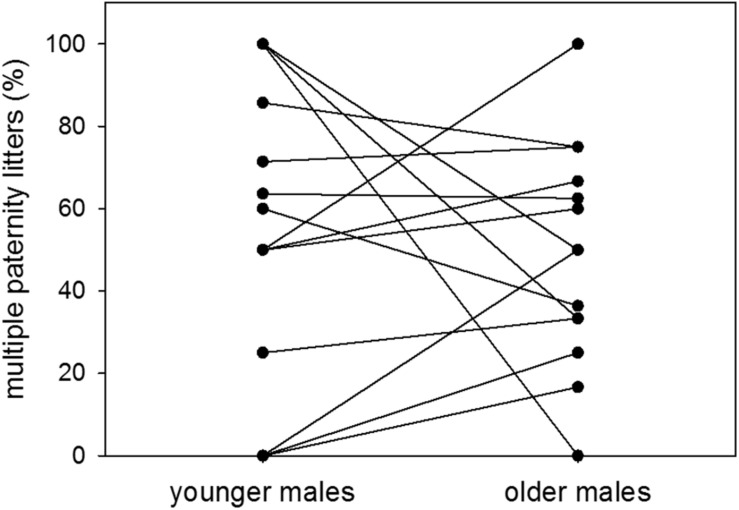
Age and multiple paternities. Proportions (%) of multiple paternity litters to sired litters of colony-housed males in the younger (60–209 days of age) and the older (210–359 days of age) age group. Each pair of dots with connecting line represents one individual. Statistics: *n* = 14; Wilcoxon signed-rank test: *V* = 52, *p* = 0.5251.

**FIGURE 7:**
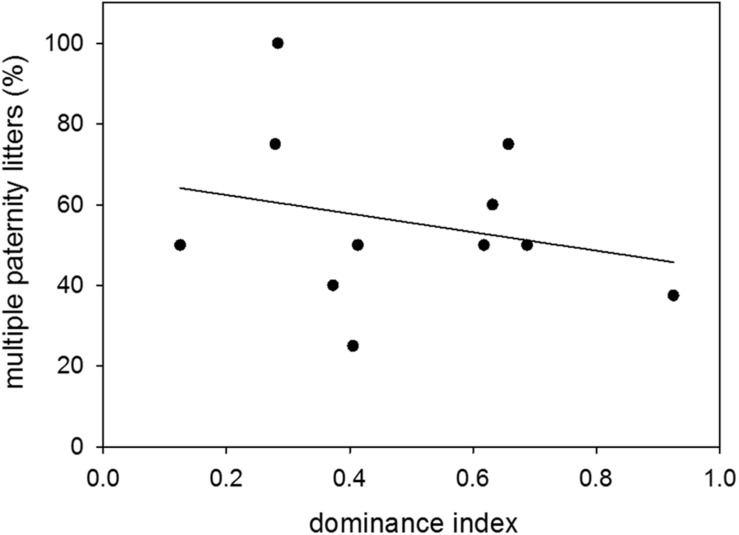
Dominance and multiple paternities. Effect of dominance index on the proportion (%) of multiple paternity litters to sired litters. Data were based on observations of colony-housed males (60–359 days of age) over about 4 months. Statistics: *n* = 11; LM: *t* = −0.78, *p* = 0.4563.

Proportions of multiple paternity litters were 54 and 49% of sired litters when males were younger and older, respectively. There was therefore no difference in the proportion of multiple paternity litters between the two age groups (*n* = 14; Wilcoxon signed-rank test: *V* = 52, *p* = 0.5251) (Figure 6). Further, there was no effect of dominance status on the proportion of multiple paternity litters (*n* = 11; LM: *t* = −0.78, *p* = 0.4563) (Figure 7).

## Discussion

The aim of the present study was to elucidate the socio-sexual development of males from onset of sexual maturity through first mating success until full adulthood in a complex social environment. For this purpose, reproductive success of guinea pigs living in large mixed-sex colonies was assessed during their first year of life. As a reference condition, a mixed-sex pair situation was used. The main findings were that males living in colonies sired their first offspring much later in life and showed a significantly higher variance in the time to their first mating success than pair-housed males. Furthermore, reproductive success in colonies was significantly affected by dominance status. Dominance itself was age-dependent with older males (7–12 months of age) having significantly higher dominance status than younger males (2–7 months of age). Interestingly, there were no indications of differences in male reproductive success between the two age groups.

First mating success of pair-housed males was achieved at 76 days of age on average with relatively low variance. This is in line with the literature, reporting that male guinea pigs usually reach sexual maturity around this time, indicated by a significant increase in testosterone levels between 50 and 90 days of age (Rigaudière et al., 1976; Sachser and Pröve, 1988) and coinciding with first sperm production around 55–60 days of age (Rigaudière et al., 1976). Thus, first mating success fell exactly in the period around which males became physiologically able to reproduce. At this time, partner females were already sexually mature, as first vaginal opening (estrus) occurs at an age of about 3–4 weeks (Sachser, 1994; Trillmich et al., 2006). Unless interrupted by pregnancy, female guinea pigs then show periodic estrus cycles of about 16 days (Ediger, 1976; Kaiser et al., 2010). Taken together, average age and age range of first mating success in pair-housed males could be fully explained by the onset of male sexual maturity and by the female reproductive cycle.

In contrast, colony-housed males sired their first offspring at 151 days of age on average and showed a much higher variance in timing of first mating success. The earliest time of reproduction was at day 56 of age, just around the onset of male sexual maturity (see above), similar to pair-housed males. However, most of the males reproduced much later. One influencing factor again was female reproductive physiology. In particular, even though up to 16 females were available in colonies, access was restricted by a post-partum estrus cycle. This means mating with each of the females was only possible every 63–72 days when they became receptive for a few hours immediately after giving birth (Kaiser et al., 2010). An additional factor in the colony situation, generated by competition, was dominance: higher ranking males had higher reproductive success than lower ranking males. Such a positive relationship between dominance status and reproductive success is not only in line with previous work in guinea pigs (Sachser et al., 1998), but can generally be found in a great variety of taxa including different fish, bird, mammalian, and even invertebrate species (Ellis, 1995; Clutton-Brock, 2016). Attaining high ranking positions requires the ability to compete with other males (Alberts, 2012; Zimmermann et al., 2017b). Hence, dominance rank is often associated with age, size, and weight in various species (Haley et al., 1994; Schuett, 1997; Alberts, 2012). Also previous work in guinea pigs showed that males in colonies usually do not reach alpha positions before an age of 7 months (Sachser, 1986), which is well beyond sexual maturity. In agreement with these results, older colony-housed males in the present study achieved significantly higher dominance status than younger males.

Based on these findings, we would have expected significantly higher reproductive success in older than in younger colony-housed males. Surprisingly, there was substantial mating success in both age groups and no difference between the two. How can this finding be explained? The limited or incomplete control model of reproductive skew proposes that subordinates will reproduce when capacity of dominant individuals to monopolize reproduction is reduced (Clutton-Brock, 1998; Reeve et al., 1998). For example, increasing individual numbers in general and of male competitors in particular, can largely impede the control of reproduction by dominant males within a group (van Noordwijk and van Schaik, 2004; Spong et al., 2008). In the present study, with up to 28 individuals and a total of 12 males per colony, group sizes were probably also too large for an effective guarding of receptive females by the most dominant alpha males. Moreover, group composition and instability of the dominance hierarchy can further play a role in diminishing reproductive skew (Alberts et al., 2003; Spong et al., 2008). In our colonies, group composition was changed about every 6 weeks by removing old and adding young individuals, most likely also causing fluctuations in dominance relationships. Likewise, these factors might have decreased reproductive skew allowing for more mating opportunities in young subordinates.

But how exactly did young males realize reproductive success? High proportions of multiple paternities of about 55% in younger males suggest that stealing copulations was very common. In general, such sneaking is a male alternative reproductive tactic that can be found in many different species (Taborsky et al., 2008; Clutton-Brock, 2016) including other highly social rodents (Schradin et al., 2012). Therefore, it seems likely that younger males applied this tactic before reaching alpha positions. Interestingly, proportions of multiple paternity litters were neither dependent on age, as older males showed similar levels of about 50%, nor dependent on dominance status. Both findings further suggest a situation of “incomplete control” by alpha males. Besides sneaking, a second possible reproductive tactic of younger males was to fight and compete with other males and to achieve mating success via dominance. Indeed, although on average higher in older males, dominance indices of younger and older animals showed substantial overlap. Thus, it seems as if mating success appears to be frequent already during the process of attaining high ranking positions, and therefore before 7 months of age. A third tactic of younger males might have been to invest in relations with particular females. Under the complex conditions of the present study, the occurrence of female choice has been already described (Sachser, 1986). As females sometimes favor younger males that are directing high amounts of courtship behavior toward them (Sachser, 1986), this might have been another way to enhance reproductive opportunities.

Generally, the three proposed reproductive tactics are probably not fixed, but rather dynamically chosen options. As already shown in other species, life-histories can be diverse and flexible, and reproductive tactics may be switched more than once during a life time (Taborsky et al., 2008; Clutton-Brock, 2016); a phenomenon referred to as social flexibility (Schradin et al., 2012). Thus, the substantial mating success before reaching a high social status was probably due to tactics flexibly applied by males already with the onset of sexual maturity.

Irrespective of the applied reproductive tactics, males in colonies generally develop a low-aggressive phenotype over the course of adolescence to preclude costly agonistic encounters with older dominant males, as has been shown by a series of experiments (see section “Introduction”; Sachser et al., 2013, 2011). Consequently, younger males were on the one hand physiologically able to reproduce but on the other hand utilized a queuing tactic. Data of the present study show clearly that younger males reproduced irrespective of queuing. In addition, reproductive success achieved by males this way was far from just “making the best of a bad job” (Schradin et al., 2009). Taken together, socially queuing but still trying to mate whenever a female was in estrus seems the optimal way for maturing males to maximize fitness in this complex social situation.

One might argue that once an individual reaches sexual maturity that further behavioral adjustment to a more distant future would be superfluous. Our results, however, show that this need not be the case. Rather, the view that substantial behavioral plasticity exists well beyond sexual maturity (Fawcett and Frankenhuis, 2015; Sachser et al., 2018) is supported by the present findings. We hypothesize that this represents a general mechanism, not only to be found in guinea pigs but also in other social mammals. Thus, for males in complex social situations, the period from reaching sexual maturity until the establishment of a stable social role might be a time during which flexible reproductive tactics are employed more commonly than usually presumed.

## Data Availability Statement

The raw data supporting the conclusions of this article will be made available by the authors, without undue reservation, to any qualified researcher.

## Ethics Statement

All procedures complied with the regulations covering animal experimentation within Germany (Animal Welfare Act) and the EU (European Community Council Directive 2010/63/EU) and were approved by the local authorities (Gesundheits- und Veterinäramt Münster, Nordrhein-Westfalen).

## Author Contributions

SK and NS conceived the study, designed the experiment, and supervised the work. AM, CR, and TZ collected the data. SK, AM, CR, SR, NS, and TZ analyzed and interpreted the data. SF and JK performed all statistical analyses. AM prepared the initial draft of the manuscript and created figures. All authors critically revised the manuscript and gave final approval for publication.

## Conflict of Interest

The authors declare that the research was conducted in the absence of any commercial or financial relationships that could be construed as a potential conflict of interest.

## References

[B1] Alberts S. C. (2012). “Magnitude and sources of variation in male reproductive performance,” in *The Evolution of Primate Societies*, eds Mitani J. C. Call J. Kappeler P. M. Palombit R. A. Silk J. B. S. (Chicago, IL: University of Chicago Press), 412–431.

[B2] Alberts S. C. Watts H. E. Altmann J. (2003). Queuing and queue-jumping: long-term patterns of reproductive skew in male savannah baboons, *Papio cynocephalus*. *Anim. Behav.* 65 821–840. 10.1006/anbe.2003.2106

[B3] Asher M. Lippmann T. Epplen J. T. Kraus C. Trillmich F. Sachser N. (2008). Large males dominate: ecology, social organization, and mating system of wild cavies, the ancestors of the guinea pig. *Behav. Ecol. Sociobiol.* 62 1509–1521. 10.1007/s00265-008-0580-x

[B4] Asher M. Spinelli de Oliveira E. Sachser N. (2004). Social system and spatial organization of wild guinea pigs (*Cavia aperea*) in a natural population. *J. Mammal.* 85 788–796. 10.1644/BNS-012

[B5] Benjamini Y. Hochberg Y. (1995). Controlling the false discovery rate: a practical and powerful approach to multiple testing. *J. R. Stat. Soc. Ser. B* 57 289–300. 10.1111/j.2517-6161.1995.tb02031.x

[B6] Bradley B. J. Robbins M. M. Williamson E. A. Steklis H. D. Steklis N. G. Eckhardt N. (2005). Mountain gorilla tug-of-war: silverbacks have limited control over reproduction in multimale groups. *Proc. Natl. Acad. Sci. U.S.A.* 102 9418–9423. 10.1073/pnas.0502019102 15964984 PMC1166604

[B7] Charpentier M. Peignot P. Hossaert-McKey M. Gimenez O. Setchell J. M. Wickings E. J. (2005). Constraints on control: factors influencing reproductive success in male mandrills (*Mandrillus sphinx*). *Behav. Ecol.* 16 614–623. 10.1093/beheco/ari034

[B8] Clutton-Brock T. H. (1998). Reproductive skew, concessions and limited control. *Trends Ecol. Evol.* 13 288–292. 10.1016/s0169-5347(98)01402-5 21238306

[B9] Clutton-Brock T. H. (2016). *Mammal Societies.* Chichester: Wiley Blackwell.

[B10] Clutton-Brock T. H. Albon S. D. (1979). The roaring of red deer and the evolution of honest advertisement. *Behaviour* 69 145–170. 10.1163/156853979x00449

[B11] Dimitrienko A. Tamhane A. C. Bretz F. (2010). *Multiple Testing Problems in Pharmaceutical Statistics.* Boca Raton, FL: Chapman & Hall.

[B12] East M. L. Hofer H. (2002). Male spotted hyenas (*Crocuta crocuta*) queue for status in social groups dominated by females. *Behav. Ecol.* 12 558–568. 10.1093/beheco/12.5.558

[B13] Ediger R. D. (1976). “Care and management,” in *The Biology of the Guinea Pig*, eds Wagner J. E. Manning P. J. (New York, NY: Academic Press), 5–12.

[B14] Ellis L. (1995). Dominance and reproductive success among nonhuman animals: a cross-species comparison. *Ethol. Sociobiol.* 16 257–333. 10.1016/0162-3095(95)00050-U

[B15] Fawcett T. W. Frankenhuis W. E. (2015). Adaptive explanations for sensitive windows in development. *Front. Zool.* 12:S3. 10.1186/1742-9994-12-S1-S3 26816521 PMC4722342

[B16] Haley M. P. Deutsch C. J. Le Boeuf B. J. (1994). Size, dominance and copulatory success in northern elephant seals, *Mirounga angustirostris*. *Anim. Behav.* 48 1249–1260. 10.1006/anbe.1994.1361

[B17] Hoffman J. I. Amos W. (2005). Microsatellite genotyping errors: detection approaches, common sources and consequences for paternal exclusion. *Mol. Ecol.* 14 599–612. 10.1111/j.1365-294X.2004.02419.x 15660949

[B18] Kaiser S. Krüger C. Sachser N. (2010). “The guinea pig,” in *The Ufaw Handbook On The Care and Management of Laboratory and Other Research Animals*, eds Hubrecht R. Kirkwood J. (Chichester: Wiley-Blackwell), 380–398.

[B19] Kalinowski S. T. Taper M. L. Marshall T. C. (2007). Revising how the computer program CERVUS accommodates genotyping error increases success in paternity assignment. *Mol. Ecol.* 16 1099–1106. 10.1111/j.1365-294X.2007.03089.x 17305863

[B20] Kanitz R. Trillmich F. Bonatto S. L. (2009). Characterization of new microsatellite loci for the South-American rodents *Cavia aperea* and *Cavia magna*. *Conserv. Genet. Resour.* 1 47–50. 10.1007/s12686-009-9011-9011

[B21] Kruuk L. E. B. Slate J. Pemberton J. M. Brotherstone S. Guinness F. Clutton-Brock T. H. (2002). Antler size in red deer: heritability and selection but no evolution. *Evolution* 56 1683–1695. 10.1111/j.0014-3820.2002.tb01480.x 12353761

[B22] Lincoln G. A. (1994). “Teeth, horns and antlers: the weapons of sex,” in *The Differences Between the Sexes*, eds Short E. V. Balaban E. (Cambridge: Cambridge University Press), 131–158.

[B23] Lürzel S. Kaiser S. Krüger C. Sachser N. (2011a). Inhibiting influence of testosterone on stress responsiveness during adolescence. *Horm. Behav.* 60 691–698. 10.1016/j.yhbeh.2011.09.007 21983230

[B24] Lürzel S. Kaiser S. Sachser N. (2011b). Social interaction decreases stress responsiveness during adolescence. *Psychoneuroendocrinology* 36 1370–1377. 10.1016/j.psyneuen.2011.03.010 21493009

[B25] Lürzel S. Kaiser S. Sachser N. (2010). Social interaction, testosterone, and stress responsiveness during adolescence. *Physiol. Behav.* 99 40–46. 10.1016/j.physbeh.2009.10.005 19835897

[B26] Martin P. R. Bateson P. P. G. (2007). *Measuring Behaviour: An Introductory Guide.* Cambridge: Cambridge University Press.

[B27] Packer C. Gilbert D. A. Pusey A. E. O’Brien S. J. (1991). A molecular gentic analysis of kinship and cooperation in African lions. *Nature* 351 562–565. 10.1038/351562a0

[B28] Reeve H. K. Emlen S. T. Keller L. (1998). Reproductive sharing in animal societies: reproductive incentives or incomplete control by dominant breeders? *Behav. Ecol.* 9 267–278. 10.1093/beheco/9.3.267

[B29] Rigaudière N. Pelardy G. Robert A. Delost P. (1976). Changes in the concentrations of testosterone and androstenedione in the plasma and testis of the guinea-pig from birth to death. *J. Reprod. Fertil.* 48 291–300. 10.1530/jrf.0.0480291 994101

[B30] Sachser N. (1986). Different forms of social organization at high an low population densities in guinea pigs. *Behaviour* 97 253–272. 10.1163/156853986x00630

[B31] Sachser N. (1994). *Sozialphysiologische Untersuchungen an Hausmeerschweinchen. Gruppenstrukturen, soziale Situation und Endokrinium, Wohlergehen. Schriftenreihe in Versuchstierkunde 16.* Berlin: Paul Parey.

[B32] Sachser N. Dürschlag M. Hirzel D. (1998). Social relationships and the management of stress. *Psychoneuroendocrinology* 23 891–904. 10.1016/s0306-4530(98)00059-6 9924743

[B33] Sachser N. Hennessy M. B. Kaiser S. (2011). Adaptive modulation of behavioural profiles by social stress during early phases of life and adolescence. *Neurosci. Biobehav. Rev.* 35 1518–1533. 10.1016/j.neubiorev.2010.09.002 20854842

[B34] Sachser N. Hennessy M. B. Kaiser S. (2018). The adaptive shaping of social behavioural phenotypes during adolescence. *Biol. Lett.* 14:20180536. 10.1098/rsbl.2018.0536 30463922 PMC6283921

[B35] Sachser N. Kaiser S. Hennessy M. B. (2013). Behavioural profiles are shaped by social experience: when, how and why. *Philos. Trans. R. Soc. B* 368:20120344. 10.1098/rstb.2012.0344 23569292 PMC3638447

[B36] Sachser N. Pröve E. (1988). Plasma-Testosterone development in colony and individually housed male guinea pigs. *Ethology* 79 62–70. 10.1111/j.1439-0310.1988.tb00699.x

[B37] Schradin C. Lindholm A. K. Johannesen J. Schoepf I. Yuen C.-H. König B. (2012). Social flexibility and social evolution in mammals: a case study of the African striped mouse (*Rhabdomys pumilio*). *Mol. Ecol.* 21 541–553. 10.1111/j.1365-294X.2011.05256.x 21883591

[B38] Schradin C. Scantlebury M. Pillay N. König B. (2009). Testosterone levels in dominant sociable males are lower than in solitary roamers: physiological differences between three male reproductive tactics in a sociably flexible mammal. *Am. Nat.* 173 376–388. 10.1086/596535 19199528

[B39] Schuett G. W. (1997). Body size and agonistic experience affect dominance and mating success in male copperheads. *Anim. Behav.* 54 213–224. 10.1006/anbe.1996.0417 9268451

[B40] Spong G. F. Hodge S. J. Young A. J. Clutton-Brock T. H. (2008). Factors affecting the reproductive success of dominant male meerkats. *Mol. Ecol.* 17 2287–2299. 10.1111/j.1365-294X.2008.03734.x 18410290

[B41] Taborsky M. Oliveira R. F. Brockmann H. J. (2008). “The evolution of alternative reproductive tactics: concepts and questions,” in *Alternative Reproductive Tactics: An Integrative Approach*, eds Oliveira R. F. Taborsky M. Brockmann H. J. (Cambridge: Cambridge University Press), 1–21.

[B42] Trillmich F. Laurien-Kehnen C. Adrian A. Linke S. (2006). Age at maturity in cavies and guinea-pigs (*Cavia aperea* and *Cavia aperea* f. *porcellus*): influence of social factors. *J. Zool.* 268 285–294. 10.1111/j.1469-7998.2005.00015.x

[B43] van Noordwijk M. A. van Schaik C. P. (2004). “Sexual selection and the careers of primate males: paternity concentration, dominance-acquisition tactics and transfer decisions,” in *Sexual Selection in Primates: New and Comparative Perspectives*, eds Kappeler P. M. van Schaik C. P. (New York, NY: Cambridge University Press), 208–229. 10.1017/cbo9780511542459.014

[B44] Wiley R. H. Rabenold K. N. (1984). The evolution of cooperative breeding by delayed reciprocity and queuing for favorable social positions. *Evolution* 38 609–621. 10.1111/j.1558-5646.1984.tb00326.x 28555990

[B45] Zimmermann T. D. Kaiser S. Hennessy M. B. Sachser N. (2017a). Adaptive shaping of the behavioural and neuroendocrine phenotype during adolescence. *Proc. R. Soc. B* 284:20162784. 10.1098/rspb.2016.2784 28202817 PMC5326539

[B46] Zimmermann T. D. Kaiser S. Sachser N. (2017b). The adaptiveness of a queuing strategy shaped by social experiences during adolescence. *Physiol. Behav.* 181 29–37. 10.1016/j.physbeh.2017.08.025 28859878

