# Supplementary material for: High Reproductive Success Despite Queuing – Socio-Sexual Development of Males in a Complex Social Environment
Source: Front Psychol. 2019 Dec 17;10:2810. doi: 10.3389/fpsyg.2019.02810 (PMC6928119; doi:10.3389/fpsyg.2019.02810)
Supplement: Supplementary file 1 [file Table_1.docx]

Supplementary Material

# Methods

## Statistics

For H1 and H2 the following model was used: Let $Y_{ij}$ be the age of first mating success of male $j$ in group $i.$ Then

$$Y_{ij}=\left\{ \begin{matrix} \tau_{P}+\epsilon_{ij} & for pair housing, i=1 \\ \tau_{C}+g_{i}+h_{ij}+\epsilon_{ij} & for colony housing, i=2,\ldots,5 \end{matrix} \right.$$

Here $\tau_{P}$ is the mean for pair housing and $\tau_{C}$ is the mean for colony housing. To allow for the fact that individuals within one colony cannot be considered independent, $g_{i}$ is the random effect of colony $i, i=2,\ldots,5$ with variance $\sigma_{g}^{2}$, while $h_{ij}$ is a random interaction between colony $i$ and individual $j$ with variance$\sigma_{h}^{2}$. The residual error has variance$\sigma_{e}^{2}$. All random effects have expectation 0. This model allows that there is a different variance for individuals in pair housing than for individuals in colony housing. In this model, the first hypothesis translates to $H_{01}:\tau_{P}=\tau_{C}$, the second translates to $H_{02}:\sigma_{h}^{2}=0$. We determined estimates $\hat{\tau_{P}}, \hat{\tau_{C}}, \hat{\sigma_{e}^{2}}, \hat{\sigma_{g}^{2}}, \hat{\sigma_{h}^{2}}$, where $\hat{\sigma_{e}^{2}}$ is the empirical variance among the individuals in pair housing, $\hat{\sigma_{h}^{2}}$ is the average empirical variance within the four colonies, while $\hat{\sigma_{g}^{2}}$ is the variance between the colonies. We tested hypothesis $H_{01}$ with the test statistic

$$t=\frac{\hat{\tau_{P}}-\hat{\tau_{C}}}{\sqrt{estimated variance}}$$

where the estimated variance is derived from $\hat{\sigma_{e}^{2}}, \hat{\sigma_{g}^{2}}$ and $\hat{\sigma_{h}^{2}}$. The t-statistic is approximatively normally distributed. To test $H_{02}$ we used the test statistic $F=\frac{\hat{\sigma_{h}^{2}}}{\hat{\sigma_{e}^{2}}}$ which was approximately F-distributed.

For data of this kind, we should expect that the variance increases with the mean. Hence, an analysis of the coefficient of variation was additionally performed. This was done by testing the equality of the variances of the logarithms calculating the F test as above based on the log(*Y_ij_*) instead of the *Y_ij_*.

For H3 we used a Poisson regression, see, e.g. Hedderich and Sachs (2012, Section 8.5.1): Let $Y_{k}$ describe the reproductive success of male $k$. Then $Y_{k}$ is Poisson distributed and

${\log E(Y}_{k})= \beta_{0}+ \beta_{1}X_{k}$

where $X_{k}$ is the dominance index of male $k$. We tested the hypothesis $H_{03}:\beta_{1}=0$with the test statistic $z=\frac{\hat{\beta_{1}}}{\sqrt{\hat{var}\left( \hat{\beta_{1}} \right)}}$, where $\hat{\beta_{1}}$ is the Maximum Likelihood estimate for $\beta_{1}$ and $\sqrt{\hat{var}\left( \hat{\beta_{1}} \right)}$ is its estimated standard deviation. The statistic $z$ is approximately normally distributed. We here used the number of sired litters to describe reproductive success. For a descriptive comparison, we also analyzed the number of sired offspring in a separate analysis.

For H4 we used a Wilcoxon-Mann-Whitney test (see test problem C with $m=12>11=n$ in Büning and Trenkler [1994, p.131ff]): Let $X_{1k}$ describe the dominance index of male $k$ of an age between 60-209 days, and $X_{2l}$ describe the dominance index of male $l$ of an age between 210-359 days and assume that these variables are independent. We tested the null hypothesis whether the two distributions (age 60-209 days versus 210-359 days) are equal. The Wilcoxon-Mann-Whitney Test assumes that the dominance indices are identically distributed which would cause no problem if all the males were in a single colony. To take into account the fact that they were from four different colonies, we augmented our test procedure by a permutation test, which only permutes the individuals within each colony. For H5 we examined the difference in the variance by means of the residuals in both subgroups using a permutation test, with the same permutations as for H4.

For H6 let $X_{1k}$ describe the reproductive success of male $k$ at age between 60-209 days, and $X_{2k}$ describe the reproductive success of the same male $k$ at age between 210-359 days. We used a Wilcoxon signed-rank test (see test problem C with $n=19$ in Büning and Trenkler [1994, p.171ff]) and tested the null hypothesis whether the two distributions (age 60-209 days versus 210-359 days) are equal. We here used the quotient between number of sired litters and number of possible litters to describe reproductive success. For a descriptive comparison, we also analyzed the quotient between number of sired offspring and number of possible offspring in a separate analysis.

For H7 we used a Wilcoxon signed-rank test (see test problem B with $n=14$ in Büning and Trenkler [1994, p.171ff]). Let $X_{1k}$ describe the percentage of multiple paternities of male $k$ at age between 60-209 days, and $X_{2k}$ describe the percentage of multiple paternities of the same male $k$ at age between 210-359 days. We tested the null hypothesis whether the two distributions (age 60-209 days versus 210-359 days) are equal. For H8 we used a linear regression and tested whether the dominance index has an impact on the proportion of multiple paternities.

H3 to H8 were tested only for colony-housed males.

**References**

Büning, H., and Trenkler, G. (1994). *Nichtparametrische Statistische Methoden*. 2nd ed. Berlin: De Gruyter.

Hedderich, J., and Sachs, L. (2012). *Angewandte Statistik*. 14. Auflage. Berlin: Springer.
